# High homocysteine levels prevent *via* H_2_S the CoCl_2_‐induced alteration of lymphocyte viability

**DOI:** 10.1111/jcmm.12829

**Published:** 2016-04-08

**Authors:** Laurie Bruzzese, Emmanuel Fenouillet, Julien Fromonot, Josée‐Martine Durand‐Gorde, Jocelyne Condo, Nathalie Kipson, Giovanna Mottola, Pierre Deharo, Régis Guieu, Jean Ruf

**Affiliations:** ^1^ UMR‐MD2Marseille Medical SchoolAix‐Marseille University and IRBANorthern sectorMarseilleFrance; ^2^ CNRSInstitut des Sciences BiologiquesFrance; ^3^ Laboratory of BiochemistryTimone University HospitalMarseilleFrance; ^4^ Cardiology DepartmentTimone University HospitalMarseilleFrance; ^5^ INSERMFrance

**Keywords:** adenosine, A_2A_ receptor, CoCl_2_, H_2_S, homocysteine, hypoxia, lymphocyte

## Abstract

High homocysteine (HCy) levels are associated with lymphocyte‐mediated inflammatory responses that are sometimes in turn related to hypoxia. Because adenosine is a potent lymphocyte suppressor produced in hypoxic conditions and shares metabolic pathways with HCy, we addressed the influence of high HCy levels on the hypoxia‐induced, adenosine‐mediated, alteration of lymphocyte viability. We treated mitogen‐stimulated human lymphocytes isolated from healthy individuals and the human lymphoma T‐cell line CEM with cobalt chloride (CoCl_2_)to reproduce hypoxia. We found that CoCl_2_‐altered cell viability was dose‐dependently reversed using HCy. In turn, the HCy effect was inhibited using DL‐propargylglycine, a specific inhibitor of the hydrogen sulphide (H_2_S)‐synthesizing enzyme cystathionine‐γ‐lyase involved in HCy catabolism. We then addressed the intracellular metabolic pathway of adenosine and HCy, and the role of the adenosine A_2A_ receptor (A_2_ _A_R). We observed that: (*i*) hypoxic conditions lowered the intracellular concentration of HCy by increasing adenosine production, which resulted in high A_2_ _A_R expression and 3′, 5′‐cyclic adenosine monophosphate production; (*ii*) increasing intracellular HCy concentration reversed the hypoxia‐induced adenosinergic signalling despite high adenosine concentration by promoting both S‐adenosylhomocysteine and H_2_S production; (*iii*) DL‐propargylglycine that inhibits H_2_S production abolished the HCy effect. Together, these data suggest that high HCy levels prevent, *via* H_2_S production and the resulting down‐regulation of A_2_ _A_R expression, the hypoxia‐induced adenosinergic alteration of lymphocyte viability. We point out the relevance of these mechanisms in the pathophysiology of cardiovascular diseases.

## Introduction

Homocysteine (HCy) is a thiol‐containing amino‐acid intermediate produced during the synthesis of cysteine from methionine 1. High HCy concentrations are associated with lymphocyte‐mediated inflammatory responses driven by immune mediators 2, 3, 4. HCy metabolism is also linked to the metabolism of adenosine 5, 6, a nucleoside that potently alters lymphocyte viability *via* activation of its A_2A_ receptor (A_2A_R) 7. Adenosine has therefore an anti‐inflammatory activity 8 particularly during hypoxia/ischaemia where it is produced in large amounts at the sites of injury 9.

Hyperhomocysteinaemia results from genetic enzymatic deficiencies and/or nutritional defects that affect HCy metabolism 10. Under basal conditions, adenosine and HCy result from hydrolysis of *S*‐adenosylhomocysteine (SAH) *via* the SAH hydrolase, and in hyperhomocysteinaemia conditions the hydrolase reaction reverses and SAH accumulates at the expense of adenosine. Subsequently, facilitated diffusion of plasma adenosine into the cell through equilibrative nucleoside transporters 11 increases and the adenosine depletion generated by this situation reduces stimulation of its cell surface receptors 12. This situation could contribute to pathological processes 5 by interfering, for example, with cardioprotective, vasodilatory effects resulting from A_2A_R activation 13, 14 and several epidemiological studies showed that high concentrations of HCy are indeed associated with cardiovascular diseases 15, 16, 17.

Hydrogen sulphide, an end product of HCy catabolism *via* the transsulfuration pathway, entered recently the family of gasotransmitters along with NO and CO, because of the effects of H_2_S at the cellular and molecular level 18. This gas was also considered as an autocrine/paracrine T‐lymphocyte activator 19, and we recently reported that H_2_S reverses the adenosinergic alteration of T‐lymphocyte viability *via* repression of the NF‐κB**,** which down‐regulates A_2A_R expression 20.

Based on this finding and on the literature reported above, we undertook to delineate the relationship between HCy, adenosine and A_2A_R expression in hypoxic conditions with particular emphasis on the effects of H_2_S produced by high HCy levels on the hypoxia‐induced, adenosine‐mediated (hypoxia‐adenosinergic thereafter) signalling in lymphocytes.

## Materials and methods

### Peripheral blood lymphocyte (PBL) preparation

Blood samples were collected from brachial vein of three healthy donors (two males and one female, 25, 38 and 42 years old respectively) after written consent. The study methodologies conformed to the standards set by the Declaration of Helsinki. Peripheral blood lymphocyte were isolated according to manufacturer's instructions using the Vacutainer^®^ Cell Preparation Tube density gradient system (Beckton Dickinson, Franklin lakes, NJ, USA). Cells (3 × 10^6^ cells/ml) were then incubated in the Roswell Park Memorial Institute (RPMI) 1640 medium supplemented with 2 mM l‐glutamine, 10% foetal calf serum and penicillin/streptomycin (100 U/ml, 100 μg/ml) for 1 hr at 37°C under 5% CO_2_ in a 25 cm^2^ flask (10 ml/flask). Adherent monocytes were then discarded and PBL present in culture supernatant were examined using the cell viability assay described below.

### Cell viability assay

Peripheral blood lymphocyte and CEM cells, a human lymphoma CD4^+^ T‐cell line expressing A_2A_R 21, were cultured in RPMI 1640 medium as described above. Cell viability was monitored in 24‐well plates using the 3‐(4,5‐dimethyl‐2‐thiazolyl)‐2,5‐diphenyl‐2H‐tetrazolium bromide (MTT) assay. The MTT assay produces a yellowish solution that is converted to dark blue, water‐insoluble, MTT formazan by oxidoreductase enzymes of living cells 22. MTT (0.5 mg in 100 μl of PBS, pH 7.3) was added to each well containing cells (0.5 × 10^6^ cells/ml) 3 hr prior to the end of the 24‐hr incubation period in culture medium containing 50–800 μM CoCl_2_ in the presence of phorbol myristate acetate (PMA, 50 ng/ml) and phytohemagglutinin (PHA, 5 μg/ml) as previously reported 20. Using the same procedure, we tested the effect of HCy by incubating cells in a medium containing PMA + PHA and 50–400 μM HCy that were added just prior to incubation with 100 μM CoCl_2_ (*i.e*. the ED_50_ deduced from the previous experiment). In subsequent tests, and prior to the addition of CoCl_2_, cells in the culture medium containing PMA + PHA were treated using 200 μM HCy (*i.e*. the maximal effective dose) and 2.5–10 mM DL‐propargylglycine (PPG). All the reagents were titrated individually in terms of activity in preliminary experiments to determine a range of active concentrations that were not cytotoxic. After treatment, cells were pelleted (10,000 × *g* for 15 min.) and supernatants were discarded. The insoluble violet formazan crystals contained in the cell pellets were dissolved using 1 ml 100% dimethyl sulfoxide and absorbance (*A*) was measured at 550 nm.

### Cell culture experiments

CEM cells (0.5 × 10^6^ cells/ml) were seeded in 75‐cm^2^ flasks (50 ml/flask) and stimulated using PMA + PHA as above. The hypoxic injury was achieved by adding 100 μM CoCl_2_ to the culture medium for 24 hr. To test the effect of HCy, cells were treated by adding 200 μM HCy just prior to the addition of CoCl_2_. To test the effect of 10 mM PPG on cells treated with HCy, the reagent was added concomitantly to HCy. In preliminary experiments, effective doses of CoCl_2_, HCy and PPG were determined using the cell viability assay described below. All the drugs were added rapidly and sequentially as follows: PMA + PHA, HCy, PPG and, *in fine*, CoCl_2_ whose presence triggered the hypoxia reaction. After 24‐hr incubation in one of the following conditions (PMA + PHA containing culture medium only, idem + CoCl_2_, +CoCl_2_ and HCy, +CoCl_2_, HCy and PPG), living cells were counted using the Trypan Blue dye exclusion method, aliquoted and centrifuged (1000 × *g* for 5 min.). Cell pellets were then frozen at −80°C until use. All reagents were from Sigma‐Aldrich (St. Louis, MO, USA).

### A_2A_R expression

A_2A_R expression was assessed using a semi‐quantitative Western blot procedure described previously 23. Briefly, cell pellets were lysed using a 4% sodium dodecyl sulphate aqueous solution supplemented with a protease inhibitor cocktail (Sigma‐Aldrich) and a 15 min.‐sonication treatment at 47 kHz. Cell lysates (0.5 × 10^6^) were diluted in loading buffer (65.2 mM Tris‐HCl buffer, pH 8.3, containing 10% glycerol, 0.01% bromophenol blue and 5% 2‐mercaptoethanol). Samples were then analysed using 12% sodium dodecyl sulphate‐polyacrylamide gel electrophoresis and electroblotting onto polyvinylidene difluoride membrane. Blots were incubated for 20 min. with an anti‐A_2A_R mouse mAb (Adonis; 1 μg/ml) 24; an anti‐β actin mAb (clone AC‐15, Sigma‐Aldrich; 0.5 μg/ml) was used as total protein loading control. Blots were then processed using horseradish peroxidase‐labelled antimouse antibodies and chemiluminescent substrate (SuperSignal West Femto; Pierce Biotechnology, Rockford, IL, USA). Densitometry analysis of the A_2A_R band at 45 kDa was performed with a Kodak Image Station 440CF (Eastman Kodak Company, Rochester, NY, USA) and the ImageJ software (NIH). Results were expressed as arbitrary units (AU) defined as pixels of one peak versus the sum of peaks (percent).

### Adenosine and inosine assays

Intracellular adenosine and inosine concentrations were measured as previously described 25 using a high performance liquid chromatography system equipped with a diode array detector (Chromsystems, Munich, Germany). To prevent adenosine degradation, pellets of frozen cells (3 × 10^6^) were mixed with 500 μl of a cold stop solution [0.2 mM dipyridamole, 4.2 mM ethylenediaminetetraacetic acid, 5 mM erythro‐9‐(2‐hydroxy‐3‐nonyl) adenine, 79 mM α‐β methylene adenosine 5′diphosphates and 1 IU/mL heparin sulphate in NaCl 0.9%]. Proteins were precipitated using 1 N perchloric acid and the supernatant was injected into a LiChrospher C18 column (Merck, Darmstad, Germany). Adenosine and inosine were identified in the same run by their elution time and spectrum. Measurements were made by comparison of peak areas versus those obtained using adenosine and inosine standard solutions. The intra‐assay and interassay coefficients of variation ranged from 1% to 3% for both products.

### Adenosine deaminase (ADA) activity

Adenosine deaminase activity was measured as previously described 23. Briefly, cells (1.5 × 10^6^) were lysed using 250 μl of ultra‐pure water, mixed with 750 μl of 28 mM adenosine in 0.9% NaCl and incubated for 36 min. at 37°C. The reaction was stopped by immersion of the samples in ice water. Ammonium production resulting from adenosine degradation by ADA was determined using a Synchron LX 20 analyser (Beckman Coulter Inc., Villepinte, France). The intra‐assay and interassay coefficients of variation ranged from 3% to 5%.

### HCy and SAH assays

HCy and SAH concentrations in cell pellets were measured using dedicated ELISA kits (Cusabio, Wuhan Huamei Biotech Co., Ltd, Wuhan, Hubei Province, China). These assays use a quantitative sandwich enzyme immunoassay technique where HCy or SAH are trapped onto an antibody‐coated plate prior to detection using a biotin‐conjugated antibody specific for HCy or SAH and an avidin‐conjugated horseradish peroxidase. For these assays, 2.5 × 10^6^ and 0.5 × 10^6^ cells for HCy and SAH, respectively, were lysed using 100 μl of 0.25% dodecyl trimethyl ammonium bromide in the assay buffer prior to transfer to microplate according to manufacturer's instructions.

### H_2_S assay

H_2_S production by cells was measured using the methylene blue method following trapping of the sulphides present in the culture medium using zinc acetate in alkaline conditions 26. Briefly, culture supernatants (50 ml; 0.5 × 10^6^ cells/ml) were mixed with 0.5 g of Zn acetate and 630 μl NaOH 10 N prior to centrifugation at 3000 × *g* for 10 min. The supernatants were removed by decantation and the Zn sulphide pellets were washed once with 25 ml ultra‐pure water. The pellets were then suspended in 1 ml ultra‐pure water and vigorously shaken. One hundred microlitres of 20 mM N,N dimethyl‐*p*‐phenylenediamine sulphate in 7.2 N HCl was then added followed by 100 μl of 30 mM FeCl_3_ in 1.2 N HCl and the tubes were shaken again. After 20 min. incubation at room temperature in the dark, the samples were centrifuged and the resulting methylene blue dye in the supernatant was measured at 670 nm. The calibration curve of absorbance versus sulphide concentration was obtained using known concentrations of NaHS, a H_2_S donor. The H_2_S concentration was taken as 30% of the NaHS concentration in the calculation 27.

### 3′, 5′‐cyclic AMP (cAMP) assay

The concentrations of cAMP present in cell pellets were measured using the Amersham Biotrak kit (GE Healthcare Life Sciences, Buckinghamshire, UK) that combines the use of a peroxidase‐labelled cAMP conjugate and a specific antiserum immobilized on microplates. Cells (1 × 10^6^) were lysed using 100 μl of 0.25% dodecyl trimethyl ammonium bromide in the assay buffer prior to transfer to microplate wells to carry out the competitive enzyme immunoassay according to manufacturer's instructions.

### Statistical analysis

Each assay performed in duplicate was repeated three times. Data (mean ± S.D.) were compared using the one‐way anova with the Fisher's least significant difference test. Differences with *P* < 0.05 were considered to be statistically significant.

## Results

### HCy prevents the hypoxia‐induced alteration of lymphocyte viability

**Figure 1:**
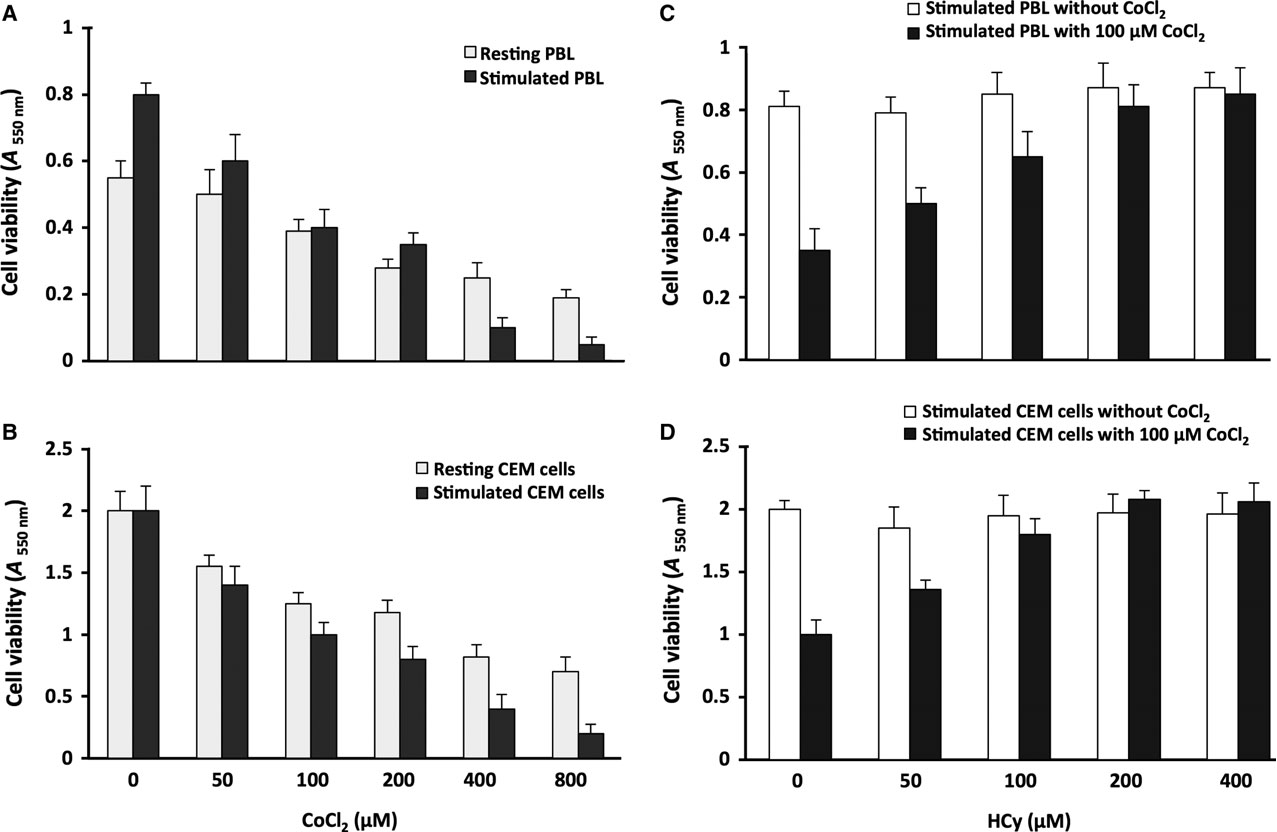
Cell viability following CoCl_2_‐ and HCy‐treatments. The viability index of PBL (**A**) and CEM cells (B), resting or stimulated using PMA+PHA and incubated with 50–800 μM CoCl_2_ for 24 hr was monitored using the MTT assay. Incubation with 50–400 μM HCy in the presence, or not, of 100 μM CoCl_2_ was similarly tested using stimulated PBL (**C**) and CEM cells (B). The dark blue dye produced by living cells was spectrophotometrically measured. Data are given as *A*_550 nm_ value (mean ± S.D., *n* = 3).

Freshly isolated PBL were used to address the authentic cell situation, whereas the human lymphoma T‐cell line CEM was used because it constitutes a cell material whose characteristics are well documented and stable. We mimicked classically the inflammatory context using PMA (5 μg/ml) and PHA (50 ng/ml), and we reproduced the hypoxia‐induced effects using a 50–800 μM CoCl_2_ treatment, a widely used method preventing HIF‐1α degradation 28, 29 that was recently validated in much detail in this exact cellular model 20. CoCl_2_ also mimics hypoxia/ischaemia responses by inducing oxidative stress and inflammation 30, 31, 32, 33. Various parameters (cell density, CoCl_2_ concentration, treatment duration) were tested in preliminary experiments (data not shown) using the MTT assay to determine the conditions used subsequently. We then choose a low cell density (0.5 × 10^6^ cells/ml) to avoid cell confluency during the experiment, and a long time exposure (24 hr) to drugs (CoCl_2_, HCy, PPG) to mimic a chronic situation. As previously obtained with more cells and a shorter time 20. Figure 1 shows that CoCl_2_ treatment affected in a dose‐dependent manner the viability of resting PBL (Fig. 1A) and CEM cells (Fig. 1B), PMA + PHA stimulation further increasing the CoCl_2_ effect. We next tested the effect of 50–400 μM HCy on lymphocyte viability in a PMA + PHA‐containing culture medium in the presence, or in the absence, of CoCl_2_. A 100 μM CoCl_2_ concentration was chosen to mimic hypoxic conditions because it corresponded to the concentration that reproducibly induced the half‐maximal effect on stimulated PBL (Fig. 1A) and CEM cell (Fig. 1B) viability. In the absence of CoCl_2_, HCy did not affect cell viability, whereas the effect resulting from CoCl_2_ treatment for 24 hr was reversed in a dose‐dependent manner using HCy in PBL (Fig. 1C) and CEM cell cultures (Fig. 1D). We previously obtained similar results using NaHS, a H_2_S donor 20.

These data indicate that high HCy levels promoted lymphocyte survival in hypoxic conditions, and we used subsequently the maximal effective concentration (200 μM) of HCy.

### PPG modulation of HCy effect

**Figure 2:**
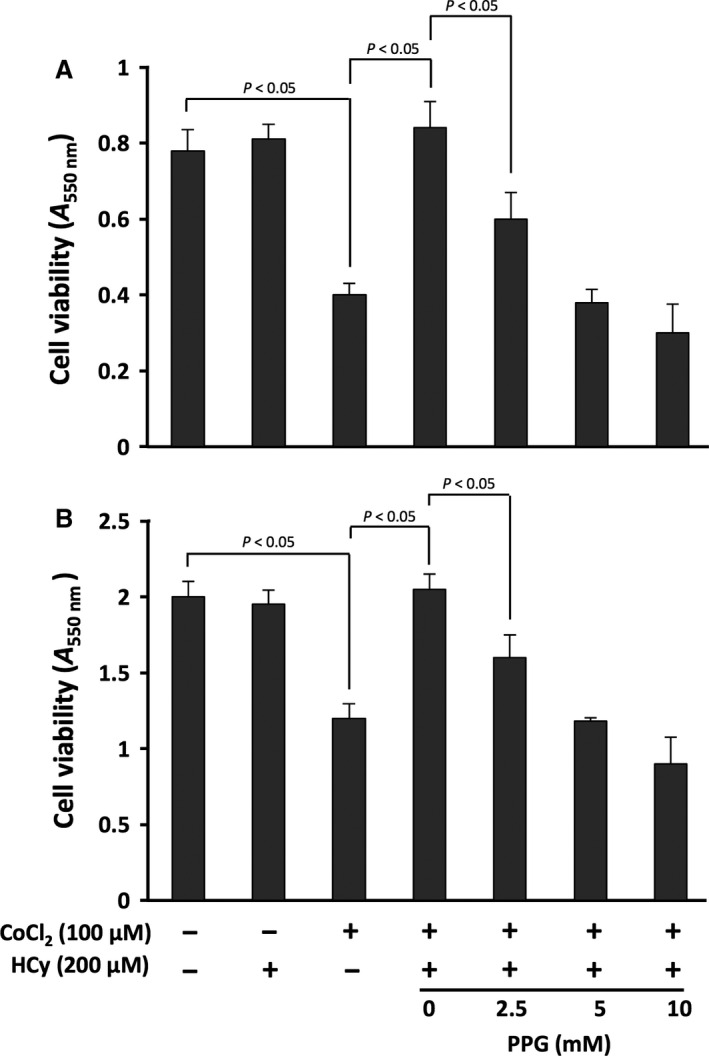
Effect of PPG in hypoxia and hyperhomocysteinaemia conditions. PMA+PHA‐stimulated PBL (**B**) and CEM cells (**B**) treated with 100 μM CoCl_2_ and 200 μM HCy for 24 hr were incubated with 2.5–10 mM PPG. The viability index was monitored using the MTT assay. Data are given as *A*_550 nm_ value (mean ± S.D., *n* = 3). Controls were cells incubated with/without 100 μM CoCl_2_ and/or 200 μM HCy. Statistical significance (*P* < 0.05) was indicated by brackets where applicable.

PPG is a specific inhibitor of cystathionine‐γ‐lyase (CSE), and consequently an agent that represses the CSE‐mediated H_2_S production 34. To address the mode of action of HCy, we examined using 2.5–10 mM PPG whether HCy acted *via* its degradation product H_2_S that down‐regulates A_2A_R expression 20. Using a high concentration (200 μM) of HCy, and in a context mimicking chronic hypoxia (24 hr incubation with 100 μM CoCl_2_), PPG treatment decreased in a dose‐dependent manner the viability of stimulated PBL (Fig. 2A) and CEM cells (Fig. 2B) versus the ‘hypoxia + hyperhomocysteinemia’ condition.

These data suggest that the effect of HCy on cell viability was reversed by CSE inhibition, and resulted therefore from H_2_S production. To further delineate this mechanism, we used in the next experiments the highest concentration of PPG (10 mM) we tested.

### Adenosine metabolism

In hypoxic conditions, adenosine is produced intracellularly from ATP and can be secreted *via* equilibrative nucleoside transporters 35. In the cell, adenosine can be degraded into inosine by ADA. Adenosine can be also produced in the extracellular environment by the ectoATPase/apyrase CD39 and ectonucleotidase CD73 36 prior to its degradation by ADA associated at the cell surface *via* CD26 37. In lymphocyte population, extracellular adenosine can be produced from ATP by the CD4^+^, CD25^+^ regulatory T cells that express both CD39 and CD73 at high levels 38.

**Figure 3:**
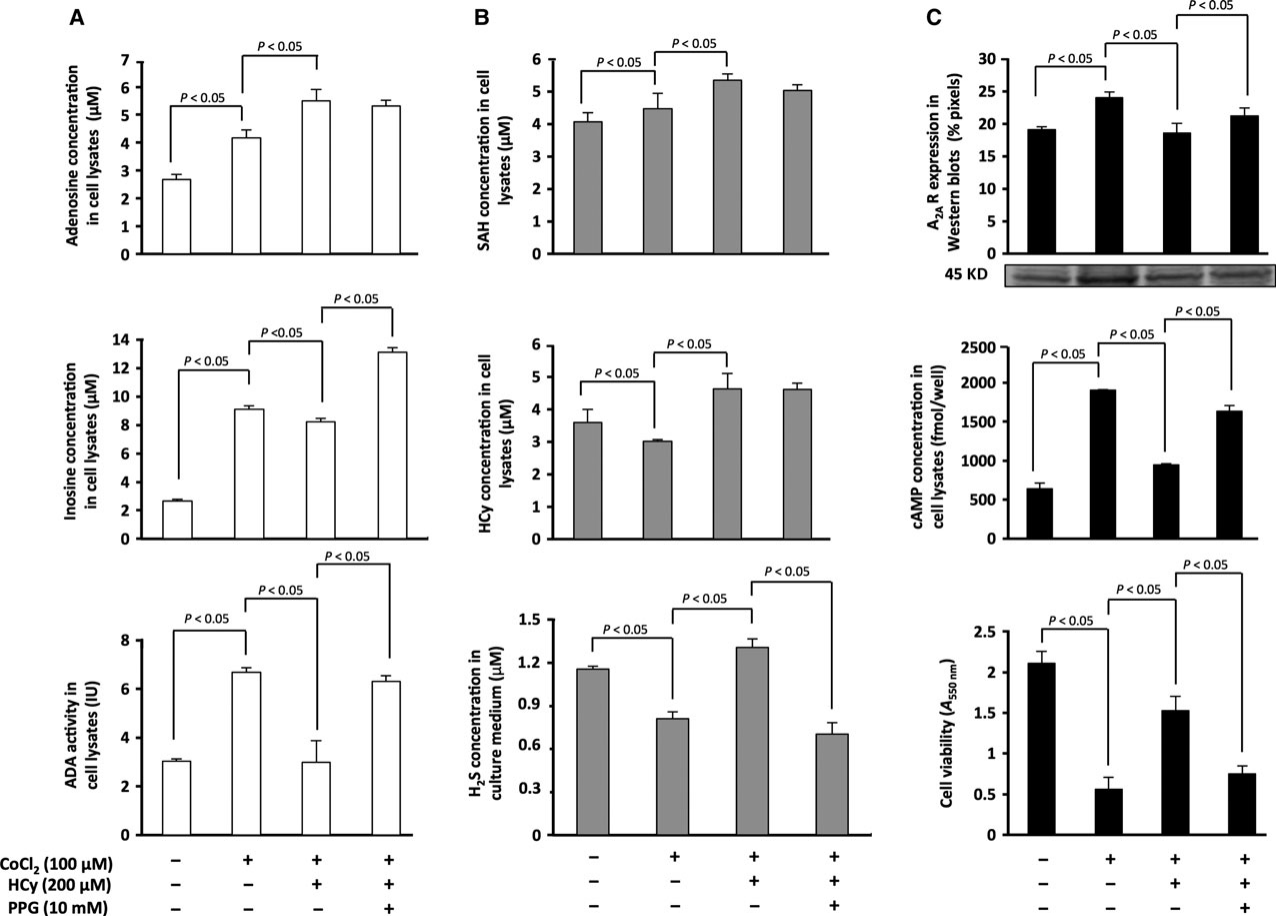
Metabolism of adenosine and HCy; adenosinergic signalling. Intracellular adenosine/inosine concentrations, as well as ADA activity, were measured in cell lysates (**A**). Intracellular SAH and HCy concentrations were measured in cell lysates, H_2_S being measured in culture supernatants (**B**). A_2_ _A_R expression, cAMP concentration and viability of the cells were also measured (**C**). PMA+PHA‐stimulated CEM cells were cultured for 24 hr in four experimental conditions: the ‘control’ condition (stimulated cells), ‘hypoxia’ (**+**100 μM CoCl_2_), ‘hypoxia+hyperhomocysteinaemia’ (**+**200 μM HCy) and ‘hypoxia + hyperhomocysteinaemia’ in the presence of 10 mM PPG. Results are mean ± S.D., *n* = 3. Statistical significance (*P* < 0.05) was indicated by brackets where applicable. Specific A_2_ _A_R bands (45 kDa) from a representative Western blot are shown in the insert.

Here, we focused on the intracellular adenosine metabolism by testing adenosine and inosine levels as well as ADA activity in lysates of stimulated CEM cells cultured in various conditions. The results are given in Figure 3A: (*i*) CoCl_2_ treatment induced a rise in cellular concentrations of adenosine and inosine (4.17 *versus* 2.68 μM and 9.09 *versus* 2.67 μM in basal conditions respectively), the latter resulting probably from the increase in ADA activity (6.68 *versus* 3.03 IU) produced to counteract the elevated adenosine production generated by hypoxia; (*ii*) a treatment combining CoCl_2_ and HCy tended to further increase the concentration of adenosine generated by hypoxia (5.50 *versus* 4.17 μM in hypoxia) while ADA activity and the resulting inosine concentration decreased (2.99 *versus* 6.68 IU and 8.23 *versus* 9.09 μM respectively); (*iii*) a treatment combining CoCl_2_ and HCy together with PPG to inhibit H_2_S produced *via* HCy catabolism did not modify adenosine concentration (5.32 *versus* 5.50 μM in the ‘hypoxia + hyperhomocysteinemia’ condition) but increased inosine production (13.10 *versus* 8.23 μM), a result which was consistent with the parallel increase in ADA activity (6.32 *versus* 2.99 IU).

These results suggested that the recovery of lymphocyte viability resulting from HCy treatment (Fig. 1C and D) was not because of a decreased production of adenosine since in fact its intracellular concentration increased (Fig. 3A).

### HCy metabolism

During the conversion of methionine to HCy, *S*‐adenosylmethionine is produced from ATP. After demethylation, SAH is generated and subsequently degraded by the SAH hydrolase into HCy and adenosine that are quickly used in basal conditions, the enzyme being also able to function in the opposite direction 39. HCy may be then remethylated to methionine or it may be condensed with serine to form cystathionine and then cysteine *via* the transsulfuration pathway 40. H_2_S, an end product of the pathway, is produced by cystathionine β‐synthase and CSE from HCy during T‐cell activation 19. In the following experiments, we focused on H_2_S production by CSE because: (*i*) CSE controls the metabolism of cysteine into H_2_S, pyruvate and NH4^+^ 41; (*ii*) CSE is the main player in hyperhomocysteinaemia conditions 42 and iii) CSE can be specifically inhibited using PPG 34.

The HCy metabolism in lymphocyte was studied by testing HCy and SAH concentrations in lysates of stimulated CEM cells cultured in various conditions while H_2_S intracellularly produced and subsequently secreted in the culture medium was measured in the culture supernatant. Our findings are given in Figure 3B: (*i*) following CoCl_2_ treatment, HCy and hence H_2_S concentration, decreased *versus* basal conditions (3.01 *versus* 3.59 μM and 0.81 *versus* 1.01 μM respectively). Conversely, SAH production increased (4.47 *versus* 4.07 μM) as a result of the hypoxia‐induced production of adenosine; (*ii*) a treatment combining 100 μM CoCl_2_ and 200 μM HCy in the culture medium markedly increased intracellular HCy concentration (4.63 *versus* 3.01 μM in hypoxia); this situation stimulated intracellular SAH production (5.34 *versus* 4.47 μM), which in turn promoted HCy degradation *via* the transsulfuration pathway into H_2_S that strongly increased in culture medium (1.31 *versus* 0.81 μM); (*iii*) a treatment combining CoCl_2_, HCy and PPG did not significantly alter the concentration of SAH and HCy (5.03 *versus* 5.34 μM and 4.61 *versus* 4.63 μM in the ‘hypoxia + hyperhomocysteinaemia’ condition respectively) but strongly decreased H_2_S production (0.71 *versus* 1.31 μM).

These data further support the conclusion that hyperhomocysteinaemia reversed the hypoxia‐adenosinergic alteration of lymphocyte viability *via* H_2_S production.

### Adenosinergic signalling

Adenosine inhibits lymphocyte viability mainly *via* A_2A_R signalling and stimulation of adenylyl‐cyclase and G_S_‐proteins 43, 44, which controls in turn a plethora of biological processes 45. We addressed here the hypoxia‐adenosinergic lymphocyte signalling by testing A_2A_R expression, cAMP production and viability of stimulated CEM cells cultured in various conditions. The data are given in Figure 3C: (*i*) CoCl_2_ treatment increased A_2A_R expression and cAMP production compared with the basal situation (24.0 *versus* 19.1 AU and 1902 *versus* 643 fmol/well respectively). This result reflected the binding of adenosine produced by the cell to an increasing number of A_2A_R, which decreased lymphocyte viability as shown using the MTT assay (*A*_550 nm_ = 0.56 *versus* 2.11 in the basal conditions); (*ii*) a treatment combining CoCl_2_ and HCy reversed the inhibitory effect on viability (*A*_550 nm_ = 1.53 *versus* 0.56 in hypoxia) by decreasing A_2A_R expression and the resulting cAMP production (18.6 *versus* 24.0 AU and 947 *versus* 1902 fmol/well respectively); (*iii*) a treatment combining CoCl_2_, HCy and PPG prevented the effect of HCy and increased A_2A_R expression as well as cAMP production (21.2 *versus* 18.6 AU and 1638 *versus* 947 fmol/well in the ‘hypoxia + hyperhomocysteinaemia’ condition respectively), cell viability decreasing (*A*_550 nm_ = 0.75 *versus* 1.53) to a value close to that found in the ‘hypoxia’ condition (*A*_550 nm_ = 0.56).

These results indicate that the loss of the hypoxia‐adenosinergic alteration of lymphocyte viability observed in the presence of HCy (Fig. 1C and D) mainly depended on the decreased level of A_2A_R expression (Fig. 3C) resulting from H_2_S production (Fig. 3B).

## Discussion

**Figure 4:**
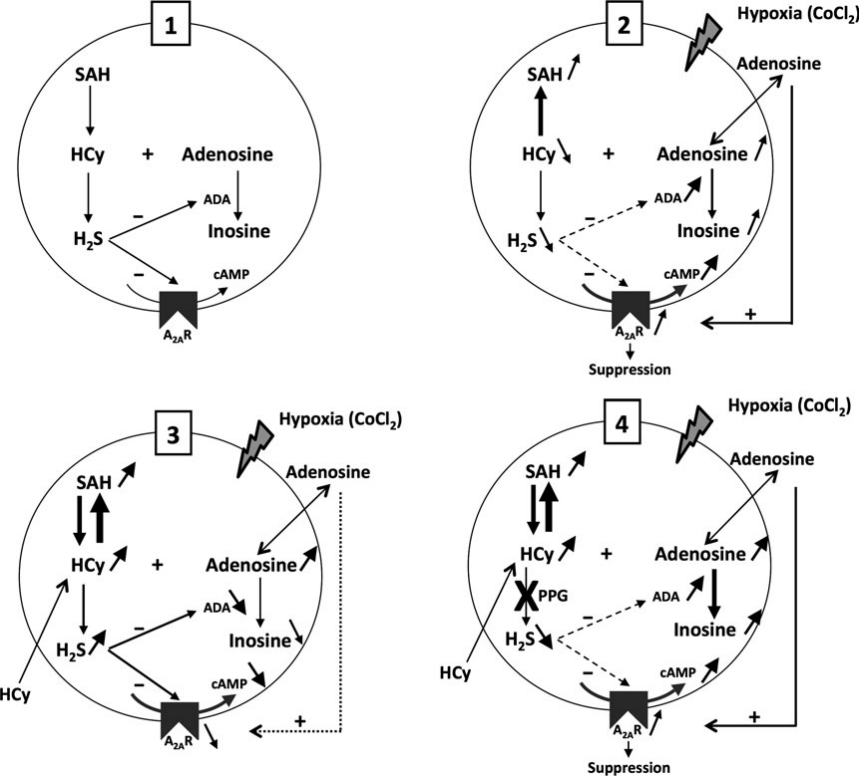
Proposed overview of HCy modulation of the adenosinergic signalling in lymphocytes. Four experimental cell culture conditions are considered: the ‘control’ condition (PMA+PHA‐stimulated cells) (1), ‘hypoxia’ (2), ‘hypoxia+hyperhomocysteinaemia’ (3) and ‘hypoxia+hyperhomocysteinaemia’ in the presence of PPG (4). Up/down arrows represent the increase/decrease in a parameter respectively. The thickness of the arrow reflects the level of the parameter. CoCl_2_‐mediated hypoxia induces intracellular production of adenosine that can exit the cell through equilibrative nucleoside transporters. Adenosine stimulates A_2_ _A_R that triggers the cAMP‐mediated suppressive signal. HCy is added to the cell medium. H_2_S is produced from HCy by CSE that is inhibited by PPG. H_2_S represses ADA production and A_2_ _A_R expression.

We found here that in CoCl_2_‐induced hypoxic conditions, high HCy levels increased H_2_S production, which down‐regulated A_2A_R expression and repressed the potent hypoxia‐adenosinergic alteration of lymphocyte viability. We performed preliminary experiments in which the monitoring of cell viability was used to dissect HCy regulation of hypoxia‐adenosinergic signalling in lymphocytes. We conducted four experimental conditions in culture medium supplemented with PMA + PHA: the control situation, hypoxia, hypoxia and hyperhomocysteinaemia, and hypoxia and hyperhomocysteinaemia in the presence of PPG. We previously reported using the same CEM cell line that hypoxia stimulates adenosinergic signalling 23 and that A_2A_R stimulation decreases lymphocyte viability 20, 21. Here, we confirmed these previous data and detailed the metabolic pathways of HCy and adenosine that are interdependent and involved in the control of lymphocyte viability (see Fig. 4 for an overview).

Firstly, we found that hypoxia increased intracellular adenosine concentration to form SAH at the expense of HCy *via* the SAH hydrolase. In basal conditions, the hydrolase tends to produce adenosine and HCy from SAH because these products are rapidly catabolized 39. Under hypoxic conditions, intracellular adenosine synthesis increases, which results in a shift of the enzymatic reaction towards SAH production from adenosine and HCy as shown here. A major consequence is that HCy is no longer catabolized to form H_2_S, and this situation prevents the known down‐regulation of adenosinergic signalling by H_2_S 20. Taking into account that lymphocytes, in particular T lymphocytes, play a pivotal role in interaction with other immune cells such as monocytes and neutrophils in an inflammatory context, repressing the lymphocyte response in hypoxic condition can contribute to the protective effect of A_2A_R activation, notably in cardiovascular disease 46. In the heart, it is also noteworthy that production of adenosine following hypoxia/ischaemia has positive effects on the vasculature *via* the A_2A_R‐mediated coronary vasodilation process 47, 48, 49. Thus, during hyperhomocysteinaemia in hypoxic/ischaemic conditions, the catabolism of HCy into H_2_S and its beneficial action on lymphocyte viability may be of particular importance regarding the onset and progression of cardiovascular disorders that generally include a strong inflammatory background 15.

Secondly, when high extracellular concentrations of HCy were added to the cell environment to mimic hyperhomocysteinaemia, HCy entered the cell by transporters similar to those for cysteine and cystine 50. In these conditions, intracellular HCy was no longer the limiting substrate for the production, together with adenosine, of SAH that strongly increased. The resulting high SAH concentration then forced the SAH hydrolase‐mediated reaction to reverse and produce, as in basal conditions, HCy and adenosine that accumulated into the cell. Excess of intracellular HCy produced from SAH could be in turn degraded to generate H_2_S that repressed both ADA activity and A_2A_R expression. The resulting low ADA activity enabled to increase intracellular adenosine concentration and to decrease inosine production, whereas the low A_2A_R expression restored the lymphocyte viability.

Thirdly, we further showed that HCy reversed the suppressive effect of hypoxia on lymphocyte viability *via* its catabolism into H_2_S, PPG preventing the H_2_S inhibitory effect on both ADA activity and A_2A_R expression. In these conditions, adenosine could then exit the cell to activate the highly expressed A_2A_R that, in turn, produced high level of cAMP as in hypoxic conditions, whereas excess of intracellular adenosine was catabolized by ADA into inosine. We observed, however, that PPG did not increase intracellular homocysteine level, suggesting that homocysteine was metabolized further into cystathionine by cystathionine β‐synthase but not into the H_2_S end product that requires the CSE activity. Moreover, the increase in H_2_S production triggered by HCy was completely abolished by PPG. These data are significant as they provide additional evidence that CSE is a central player in H_2_S production in lymphocytes 41. By showing using PPG the H_2_S‐mediated hyperhomocysteinaemia effect on the hypoxia‐adenosinergic signalling in lymphocytes, we also support here the pro‐inflammatory role attributed to H_2_S 51 as well as its repressive action on A_2A_R expression 20.

These results show that HCy down‐regulates A_2A_R expression in hypoxic conditions *via* H_2_S production. We propose that this mechanism has strong implications for immunosuppression, and particularly for patients with cardiovascular diseases and severe hyperhomocysteinaemia that was shown to be associated with inflammation and cardiovascular complications. Our findings may provide clues to explain why hyperhomocysteinaemia constitutes a risk factor in cardiovascular diseases 15, 16, 17 and may therefore help to define new therapeutic strategies.

## Author contributions

L.B. and E.F. performed the experiments, analysed the data and wrote the paper. J.M.D.G., J.C. and N.K. performed the experiments and analysed the data. J.F., G.M. and P.D. and R.G. analysed the data and wrote the paper. J.R. designed the study, planned the experiments, analysed the data and wrote the paper.

## Conflicts of interest

The authors declare that they have no conflicts of interest with the contents of this article.
